# An Integrative Structural Health Monitoring System for the Local/Global Responses of a Large-Scale Irregular Building under Construction

**DOI:** 10.3390/s130709085

**Published:** 2013-07-15

**Authors:** Hyo Seon Park, Yunah Shin, Se Woon Choi, Yousok Kim

**Affiliations:** 1 Department of Architectural Engineering, Yonsei University, 134 Shinchon-dong, Seoul 110-732, Korea; E-Mails: hspark@yonsei.ac.kr (H.S.P.); yunah@yonsei.ac.kr (Y.S.); 2 Center for Structural Health Care Technology in Buildings, Yonsei University, 134 Shinchon-dong, Seoul 110-732, Korea; E-Mail: watercloud@yonsei.ac.kr

**Keywords:** structural health monitoring, sensor network, in-construction monitoring, irregular building

## Abstract

In this study, a practical and integrative SHM system was developed and applied to a large-scale irregular building under construction, where many challenging issues exist. In the proposed sensor network, customized energy-efficient wireless sensing units (sensor nodes, repeater nodes, and master nodes) were employed and comprehensive communications from the sensor node to the remote monitoring server were conducted through wireless communications. The long-term (13-month) monitoring results recorded from a large number of sensors (75 vibrating wire strain gauges, 10 inclinometers, and three laser displacement sensors) indicated that the construction event exhibiting the largest influence on structural behavior was the removal of bents that were temporarily installed to support the free end of the cantilevered members during their construction. The safety of each member could be confirmed based on the quantitative evaluation of each response. Furthermore, it was also confirmed that the relation between these responses (*i.e.*, deflection, strain, and inclination) can provide information about the global behavior of structures induced from specific events. Analysis of the measurement results demonstrates the proposed sensor network system is capable of automatic and real-time monitoring and can be applied and utilized for both the safety evaluation and precise implementation of buildings under construction.

## 1. Introduction

Structural health monitoring (SHM) based on sensor technology has received considerable attention and has successfully replaced traditional visual inspection for damage detection and maintenance of structures subjected to various loadings such as earthquake, wind and service loadings [1–3]. Not only traditional sensors (e.g., strain sensor [4–7], displacement transducer [8,9] and accelerometer [10–12], *etc.*) but also new measuring approaches (e.g., GPS [13,14], terrestrial laser scanning [15,16], vision-based system [17,18] and laser Doppler vibrometers [19,20], *etc.*) have been employed in the field of SHM. Applications of SHM to actual buildings largely focus on measuring the responses of in-service buildings to understand their structural conditions and performance.

For buildings under construction, however, there are fewer SHM applications due to the specificity of the construction site and various restrictions, such as limited space for sensor installation and obstacles that hinder stable monitoring. SHM was applied in a building during construction in [21], where the column strain in a high-rise residential building was measured using fiber-optic sensors. Li *et al.* [22] also employed fiber Bragg grating sensors to monitor the temperature evolution history and strain variation of columns on the underground floor of an 18-story building during construction.

The structural health monitoring on the Guangzhou New TV Tower [23] is a representative case study for the intensive monitoring of an in-construction structure; 16 types of sensors were employed, and structural responses (e.g., inclination, displacement, strain, and acceleration) and environmental factors (e.g., temperature and humidity) were monitored during construction. However, these applications to the structures under construction [21–23] were primarily based on a wired sensor network, although a wireless sensor network (WSN) was also partially employed on the Guangzhou New TV Tower [23]. However, there is little research on the SHM of irregular buildings while under construction.

It is well known that wired sensor networks are expensive—not only in terms of the sensor equipment itself but also in the additional costs and space required for cable installation [24]. In the case of in-construction monitoring, these costs can increase tremendously considering the complex environmental factors in a modern construction site [25]. In addition, there is the high possibility of losing or damaging the cables during the construction process due to the movement of construction materials and the work force, creating inconveniences in maintenance and additional costs for the re-installation and repair of the equipment [26]. For a SHM system to be practical during construction, a restrictive application of SHM must be considered at the construction site to avoid interruptions in the construction process and realize stable, automatic monitoring.

As an alternative for a wired sensor network system, a WSN system based on sensor and wireless communications technology has been employed in the SHM field; the advancements in the past decade have shown the possibilities and opportunities for stable monitoring in SHM applications [27]. A WSN presents clear advantages over a wired sensor network in terms of cost, deployment, and management. These considerations are major priorities for resolving the various issues related to in-construction monitoring. However, there are also several challenging issues for the application of WSNs in terms of long-distance transmission, limited bandwidth, and long-term power supply [28,29]. Despite the disadvantages of a WSN, the use of WSNs for SHM can clearly provide strong potential and distinct advantages over wire-based sensor networks, which have definite limitations as structures become complex and large.

Based on the limitations in wired monitoring systems and the issues concerning the application of SHM for in-construction monitoring, this study develops an integrative monitoring system and applies it to a large-scale irregular structure currently under construction with three underground stories, four above-ground stories. The structural responses (such as strain, inclination, and vertical deflection) were measured using sensors consisting of 75 vibrating wire strain gauges (VWSGs), 10 inclinometers, and three laser displacement sensors over a period of 13 months.

The contribution of this research to SHM applications for in-construction monitoring is significant for several reasons: (1) customized power-efficient wireless sensor nodes (e.g., for strain, displacement, and inclination) are developed and applied to a large-scale irregular building structure under construction, where many challenging issues exist compared to the in-service or in-construction monitoring of a regularly configured building; (2) Comprehensive communications from sensor nodes to monitoring servers are executed in a wireless transmission consisting of two types of communication modules—the Industrial Scientific and Medical (ISM) band and the Code Division Multiple Access (CDMA) method. These communication modules are responsible for short- (on-site) and long- (off-site) distance communication and utilize flexible and scalable system architectures to enable a decentralized sensor network; (3) The implementation of a management software program for the collected data enables construction administrators to monitor the structural responses automatically in real-time through Internet-based terminals, such as personal computers, tablets, and mobile phones, regardless of the time and location. Finally, based on the measured global and local responses of the actual structure under construction during the 13-month period, we investigate the applicability of the proposed practical monitoring system to promptly and accurately respond in securing the safety, stability, and construction quality of the structure.

## 2. Sensor Network for In-Construction Structural Health Monitoring

**Figure 1.**
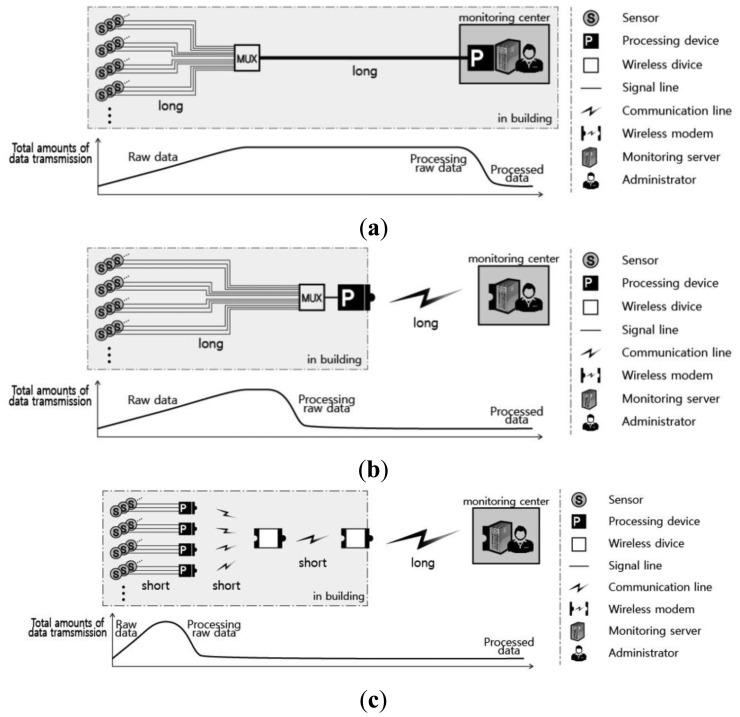
Sensor network (**a**) Wired sensor network; (**b**) Conventional wireless sensor network; (**c**) Proposed wireless sensor network.

The typical earlier forms of a SHM sensor network were wired systems, as shown in Figure 1a. There are many problems that must be addressed when using a wired network for structural monitoring during construction. The receiver of the measuring instrument (which processes the data measured by the sensor) consists of a single port, so the multiplexer (Mux) performs the role of the system multiplex, where data measured from adjacent sensors are gathered into one line. The on-site problem with applying a wired sensor network for SHM is that the entire communication between the sensors and repository responsible for data collection and processing relies on a cable that inevitably becomes too long. This long cable induces more noise into the system and is prone to interruption problems due to the high probability of wire damage resulting unexpected site conditions. Furthermore, once the cable is compromised, it is difficult to locate the damage, leading to severe drawbacks and delays in maintenance and system stability.

Recent developments in communications and sensing equipment technology have enabled the development of multifunctional wireless sensing units that are low cost, energy efficient, and small in size [30]. There are many advantages in wireless data transmission when monitoring the structural safety of a site under construction; in particular, there are fewer restrictions in installation space and considerable reductions in cable length compared to a wired sensor network when arranging the numerous sensor locations or selecting a monitoring space. In addition, with the wireless transmission of data, the monitoring server does not have to be located on site. If the length of the wireless transmission can be sufficiently extended, numerous SHM sites can be managed by simultaneously a small workforce at a dedicated control center, so multiple sites can be efficiently monitored and managed.

As shown in Figure 1b, the initial form of the WSN uses cables inside the site to collect measurement data from each sensor; these data are then wirelessly transmitted to an in- or off-site monitoring server using a wireless module [23]. However, the WSN shown in Figure 1b has a similar composition to that of the wired system (Figure 1a) within a structure or within each floor area. Therefore, the same physical problems exist in applying the system in Figure 1b as an in-construction monitoring network.

The wireless system proposed in this study refines the existing wireless monitoring system to make it more feasible. As shown in Figure 1c, a wireless transmission method is chosen for each stage to reduce the distance of the wireless transmissions, thus reducing energy consumption for the transmission and improving the energy conservation of the equipment. Furthermore, the length of the signal lines between the sensors and sensor nodes can be minimized by appropriately arranging the location of the sensors and sensor nodes. This approach has the following advantages: (i) the installation costs for cables that connect traditional sensors and wireless sensing units (sensor nodes) are significantly reduced; (ii) the interruptions to construction due to wiring installation are reduced; and (iii) the possibility of cable damage is lowered and any resulting cable damage can be promptly detected, thus reducing maintenance and management costs.

## 3. Description of the Proposed Sensor Network

### 3.1. Network Components

**Figure 2.**
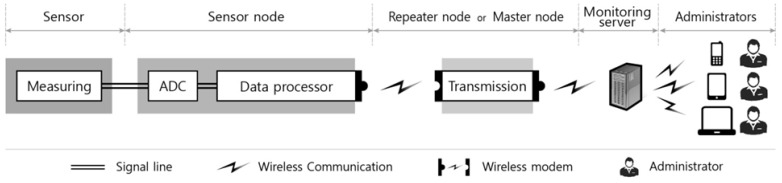
Function of each component for structural monitoring.

The composition of the monitoring system suggested in this research (which can be applied during construction) is shown in Figure 2. It consists of a sensor, a sensor node, a repeater node, a master node, and the monitoring server; the characteristics of each component are outlined below.

#### 3.1.1. Sensor

**Figure 3.**
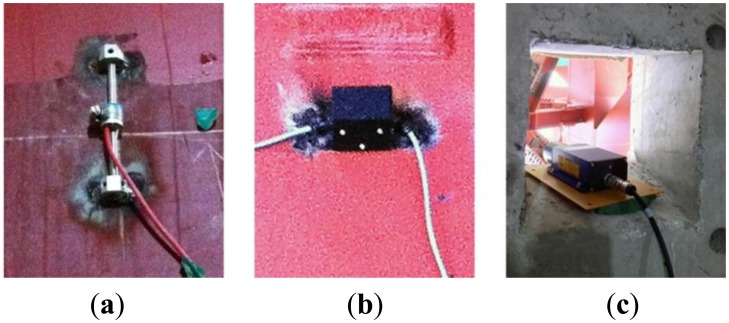
Sensors. (**a**) VWSG; (**b**) Inclinometer; (**c**) Laser displacement sensor.

As shown in Figure 3, there are three types of sensors used for measuring the structural responses in this research; a VWSG to measure strain, an inclinometer to measure inclination, and a laser displacement sensor to measure vertical deflection.

In the field of SHM, several types of strain gauges [e.g., electrical strain gauges (ESGs), fiber optic sensors (FOSs), and vibrating wire strain gauges (VWSGs)] are usually used to monitor structural responses. Among these strain-type sensors, FOSs and VWSGs, which are immune to electromagnetic interference and provide superior endurance, are actively studied and employed in SHM [31-35]. However, FOSs are extremely fragile, which contributes to a high rate of installation failure in real structures. In addition, it is impossible to avoid employment and handling of lengthy cables between the FOSs and data logging hardware. Moreover, the physical size of the data logger for VWSs is relatively smaller than that of FOSs since the measurement principle of VWSGs is very simple. Consequently, with the advantages of easy installation over other sensors, VWSGs (VSM-4000 model by Geokon, Inc. (Lebanon, New Hampshire, USA) [36]) are employed in this study.

The inclinometer (DST-MTS-V2 model by DSTEK Inc. (Seoul, Korea) [37]) and laser displacement sensor (LDS, LLD-0100 model, JENOPTIK AG, Jena, Germany, [38]) (which play important roles in precise construction monitoring) measure the structural responses of the connections or areas where excessive deflection is expected. A micro-electro-mechanical sensor biaxial inclinometer (with a thermometer) and a contactless optic laser displacement sensor were used.

The sensors automatically and continuously measure the structural responses according to pre-scheduled periods, and the measured data are transmitted to the sensor node through a signal line. Table 1 provides the specifications for the aforementioned sensors.

#### 3.1.2. Sensor Node

The raw data (e.g., voltage, strain) measured from each sensor must be converted into structural response values (e.g., displacement, strain and inclination). To obtain usable data information from the raw data, an analog-to-digital converter (ADC), responsible for signal conversion, is needed to downsize, process, and input/output the data information. In the WSN proposed here, the sensor node plays the role of the data reading device, which processes (ADC, sampling) the data transmitted from the sensors and then transmits the processed data values to a wireless transceiver (repeater node/master node). In other words, sensor node performs three functions—a sensing interface, computational core, and wireless transceiver.

Each individual sensor node is developed depending on the sensor type such as a VWSG, inclinometer, or laser displacement sensor. As a sensing interface, a VWSG sensor node is equipped with a system that drives four channels of a VWSG at each node; the inclinometer sensor node can simultaneously process a maximum of three sensors. The transmitted data passing through the signal processing of the sensor node significantly reduces the transmission amount when compared to the direct transmission of raw data. As a result, even when multiple sensors are connected to a single sensor node, this setup is more energy efficient in transmission. The inclinometer and laser displacement sensor have data processing functions included in their sensors. Therefore, the sensor nodes for the inclinometer and laser displacement sensor essentially play the role of a wireless transceiver, transmitting the sampling completed data.

#### 3.1.3. Repeater Node and Master Node

**Figure 4.**
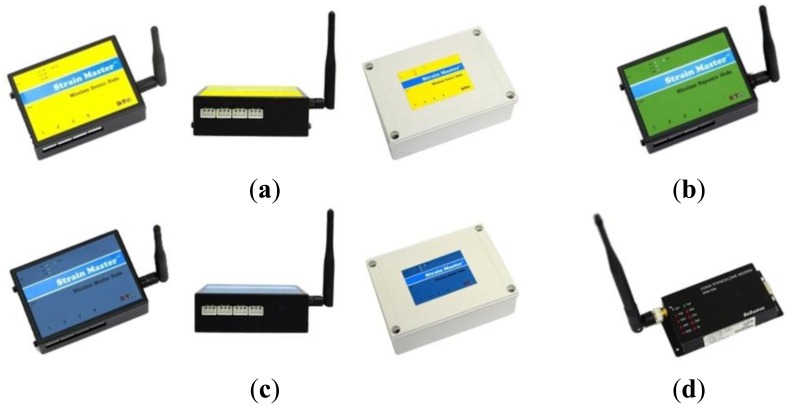
Wireless sensing units. (**a**) Sensor node; (**b**) Repeater node; (**c**) Master node; (**d**) CDMA kit.

The sensors do not possess a transmission function, so a wireless transceiver is required to send the structural response information received from each sensor to the monitoring server. In this system, the transmissions between the sensor nodes and remote server are performed by the repeater node and master node, as shown in Figure 4.

The repeater nodes have a relay function to address transmission problems that can occur in various site situations, including direct data transmissions being blocked by limited communication distances between the sensor node and master node or obstructing elements in the wireless communication. The use of repeater nodes allows the final transmission distance is extended and for stable communication.

The master node receives all of the measured data wirelessly transmitted from the sensor nodes and the repeater nodes at a short distance. Then, the master node sends the data to the remote monitoring server using long-distance communications. A single master node is able to acquire dozens of sensor data submissions, so a large amount of data transmission is possible with a small number of master nodes through wireless communications.

#### 3.1.4. Monitoring Server and User Interface Software

All of the data measured from the numerous sensors extensively installed over the monitoring site are eventually sent to the monitoring server at the monitoring control center through the master node. The monitoring control center (located far from the construction site) can manage several monitoring sites simultaneously. The system suggested in this research utilizes user interface software that can efficiently manage the received data and enable various administrators to easily check the data. This data integrated managing software program then becomes the platform for connecting multiple facilities and multiple maintenance management agents. The registration and removal of managed sites are easily accomplished, and simultaneous access from multiple administrators is possible. Software access levels can be set to limit the range of activity for each administrator.

**Figure 5.**
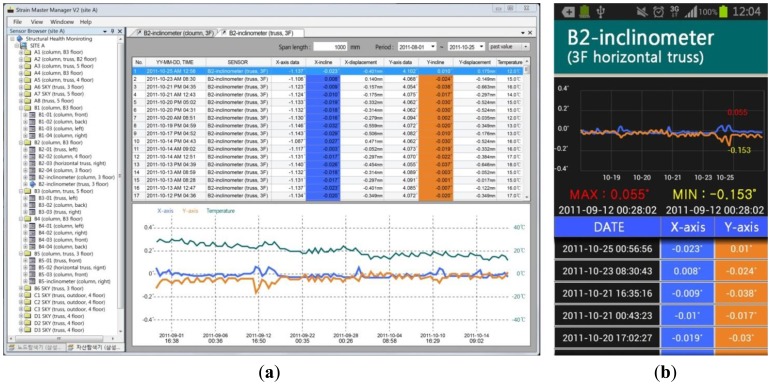
Display of the user interface software program. (**a**) Software program on the monitoring server; (**b**) Application of a mobile phone.

Figure 5a presents the monitoring software as seen by an administrator through a PC, and Figure 5b presents the application screen on a smart phone. In Figure 5a, the main menu on the top of the screen is where common information of the system is managed and where monitoring site information can be checked or modified. The sensor network of a specific site to be checked can be selected from the several monitoring sites on the browser to the left of the screen, and the zones where sensors are installed for each site are shown as a tree menu. The details regarding the specific monitoring sensor that is selected from the browser can be checked on the right side. On the upper right, the measured data transmitted from the relevant sensor can be viewed for each time period. Figure 5 presents a screenshot of strain measured at an actual site for a certain period by a inclinometer. The numerically expressed values are shown in graphical form on the lower right. Therefore, the change in measured data can be checked along with the relevant values, and any measurement data deviating from a certain range can be easily identified. Furthermore, the most recent measurements received through the real-time transmission can be checked and past measurement data can also be easily obtained by adjusting the inquiry period. In addition, the general information and battery residue information of a node can be checked by clicking on the relevant wireless node. If an administrator's contact information is entered beforehand, a warning message is sent regarding abnormalities to the wireless terminal owned by that administrator, making it possible to utilize this function to heighten response time in monitoring site management.

### 3.2. Communication Method

#### 3.2.1. From Sensor to Sensor Node

**Figure 6.**
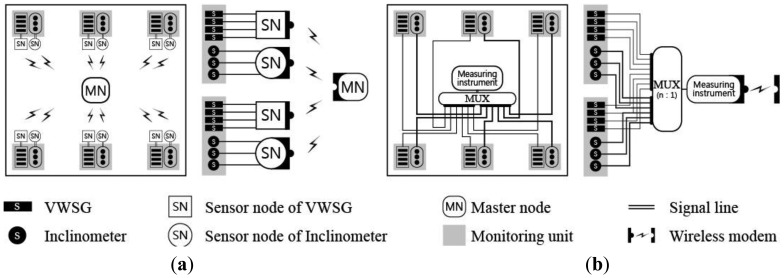
Comparison of the length of the signal line. (**a**) Wireless sensor network; (**b**) General sensor network.

The data communication from the sensor to the sensor node occurs through the signal line in this system. As shown in Figure 6a, the system suggested in this research forms a zone for sensors in close proximity, and the sensors in each zone use a different sensor node according to the sensor type. This configuration reduces both the size of the sensor node and the energy consumption while simultaneously reducing the length of the signal line to significantly lower data loss and damage. The remarkable reduction in cable length between the sensor and sensor node can be observed by comparing it to the sensor network system shown in Figure 6b.

#### 3.2.2. From Sensor Node to Master Node (via Repeater Node): ISM Band

The measured information that is transmitted from the sensor travels through the sensor node and is then transmitted to the master node through a wireless transceiver (Figure 6a). A typical construction site is comprised of structural horizontal and vertical elements, partition walls, and finishing materials and these variable large-scale members are all elements that can obstruct wireless communications. A diffractive frequency band should be used to achieve stable wireless communications among these obstructive elements. In the system proposed here, an ultrahigh frequency of 424 MHz in the ISM band was used for wireless communications between the sensor node and master node. The valid communication distance of the ISM band at 424 MHz is within 300 m when in line of sight (LOS) and within 70 m when in non-line of sight (NLOS). Compared to microfrequencies, this signal suffers less influence from interference and is more diffractive to obstructions; it can be viewed as the most appropriate wireless communication method at a construction site with various obstructions.

#### 3.2.3. From Master Node to Monitoring Server and Administrators: CDMA and the Internet

The final data transmission process—from the monitored building to the off-site monitoring control center—is performed by the master node. When all of the measured data values at the site are gathered at each master node, the information is transmitted to the monitoring server at the monitoring control center through CDMA communication, which is a digital mobile communications method using spread spectrum technology. CDMA communication is a multi-access technology that transmits coded signals to the entire available bandwidth, so multiple users can gain access in the same frequency band simultaneously. This multi-access configuration spreads the transmission of the signal to a much wider bandwidth than the bandwidth of the signal itself, so it is not interrupted by other communication sources, resulting in multiplexing and high-speed information processing. CDMA communication is resistant to interference and provides added security—the coded signal of the specific user is perceived as noise to other users. There are no distance restrictions, and there is only a low possibility of data noise due to long-distance transmissions. Furthermore, limited resources can be shared by many users, so the signal can be decoded and demodulated using wireless terminals, such as PCs, notebooks, tablets, and mobile phones, on the Internet regardless of the distance from the monitoring site. As a result, an administrator in this system can check the data for a desired location and time, and several administrators can promptly evaluate the structural safety of the site conditions regardless of time and space.

### 3.3. Low Power Consumption

The issue of a stable power supply is a crucial project priority in the stable operation of a SHM [26,27]. In wired networks, power can be supplied through a simple coaxial wire that is also responsible for communications between the sensors and server. However, in wireless sensors, a power supply typically relies on a battery with limited capacity. Therefore, it is necessary to develop a wireless sensing unit with low-power consumption and reduce the amount of data transmission through decentralization of the sensor array.

In this system, low-power technology was employed in the wireless sensing units (*i.e.*, sensor nodes, repeater nodes, and master nodes) to stabilize the operation of each component [35]. Continually operating the communication devices in a standby state to perform their data exchange functions increases the energy consumption. To solve this problem, the active mode times of the sensor node, repeater node, and master node connected to the wireless communication system are synchronized so that the sleep-active modes are independently repeated to minimize power consumption. The energy consumed during sleep mode is 170 μA, which can be sustained for long periods using only battery power. For example, a small 2,700 mAh battery can be used for 400 days or more if 24 measurements are made per day. If a solar-pack is attached as a power supply, the battery can be used indefinitely.

Because the majority of electric power is consumed during data transmission (even more than data processing), it is necessary to reduce the amount of transmitted data through transceivers (e.g., sensor node, repeater node, and master node) that are operated by limited capacity batteries. In this study, sensor groups in adjacent areas are connected to one sensor node with a signal line, through which electric power is provided. The decentralized deployment of the sensor array applied in this study reduces the number of sensors controlled by one sensor node and results in low power consumption of the sensor node.

## 4. Application of the Monitoring System

### 4.1. Target Structure

**Figure 7.**
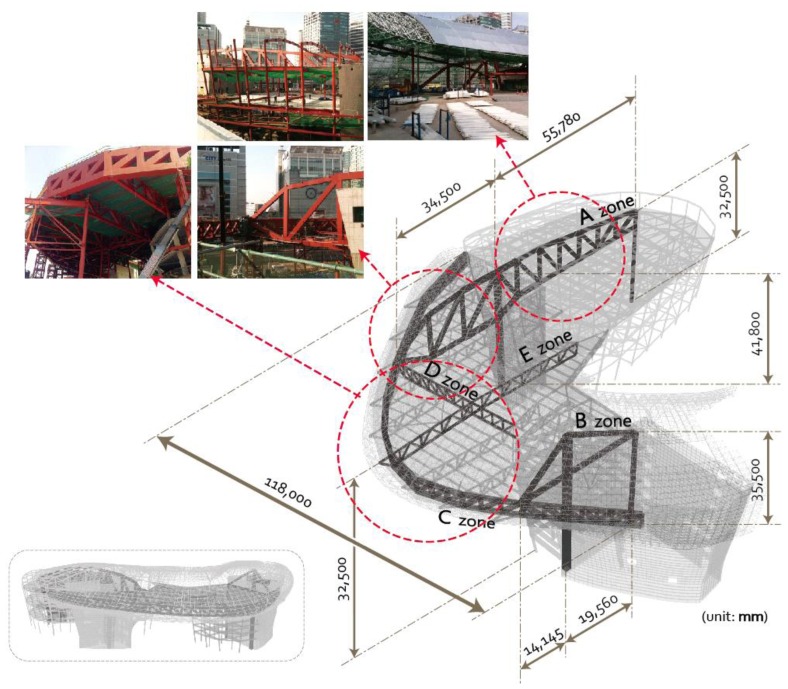
Structure zoning of SHM on the irregular shaped building at DPB.

A real-time automatic structural response monitoring system was applied to the large-scale irregular building [Design Plaza Building (DPB) in Seoul, Korea] from the stage of construction. DPB has numerous complexities in its construction such as various exposed concrete elements using free curvatures and external panels, complicated space frames supporting the panels, an irregular large internal space, and mega members. Figure 7 illustrates the framework of DPB. Zones A and B are the mega columns and mega trusses supporting the long-span edge truss with a span length of 118 m. The mega trusses and edge trusses that support this large space are cantilevered. Therefore, the vertical deflection and tilt that occurs at the free end of the cantilevered members and the stress of the various members were the primary objects for the in-construction monitoring; because of its importance, the wireless nodes were intensively installed in this location for monitoring.

**Figure 8.**
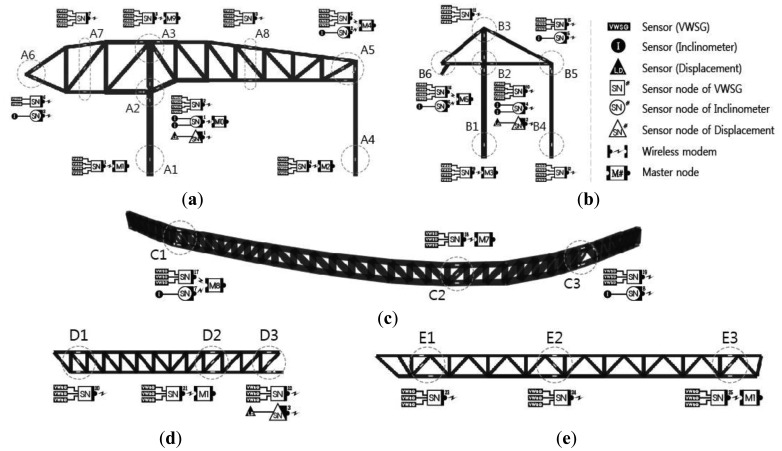
Sensing location for the monitoring zones at DPB. (**a**) A zone; (**b**) B zone; (**c**) C zone; (**d**) D zone; (**e**) E zone.

As shown in Figure 8, the structural responses, such as strain, deflection and inclination, are monitored according to the characteristics of each major structural member at DPB. Strain sensors used at DPB were installed in 75 locations at major members, such as the mega truss, mega column, edge truss, and floor truss, and measurements were performed five to six times per day. Inclinometers were installed to perform monitoring at 10 locations at the connecting areas of the mega truss, mega column, and long-span cantilever area. Excessive vertical deflection was anticipated in the long-span cantilever area; therefore, the structural responses from the vertical deflection was measured with the laser displacement sensor in three areas at the end of the cantilever in the mega truss (zone A), the end of cantilever in the mega truss (zone B), and the connecting area at the center of the edge truss (zone C).

**Figure 9.**
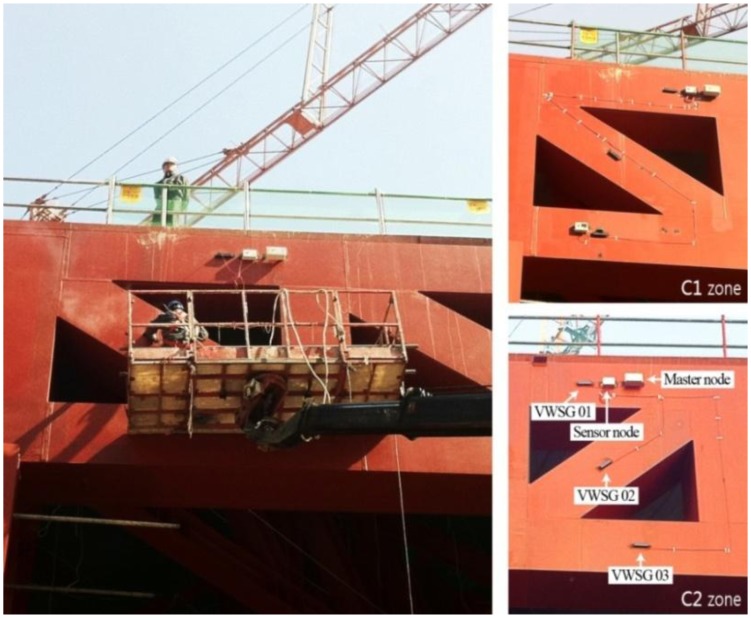
Installation of sensors in zone C.

Each sensor and wireless sensing unit was installed using a sky-vehicle or temporary structure, as shown in Figure 9.

**Figure 10.**
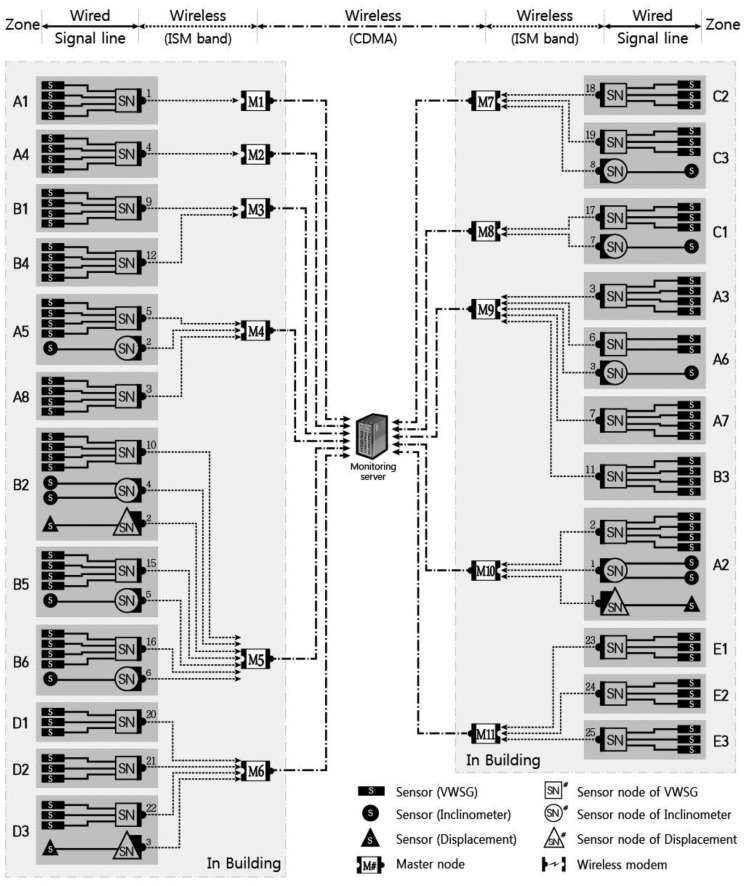
Communication network architecture at DPB.

Each sensor and sensor node was covered with a plastic cap for protection from harsh environmental conditions at the construction site. As shown in Figure 9, the signal line connecting the sensor and sensor node is very short, reducing the possibility of cable damage at the construction site. When wireless communication was impossible from the initially planned node location due to unexpected site conditions, the locations of the nodes were moved to accommodate the volatile conditions of the construction site; however, most of the wireless nodes transmitted from the locations where they were originally planned. A diagram of entire sensor network applied to DPB is presented in Figure 10.

### 4.2. Monitoring Results

**Figure 11.**
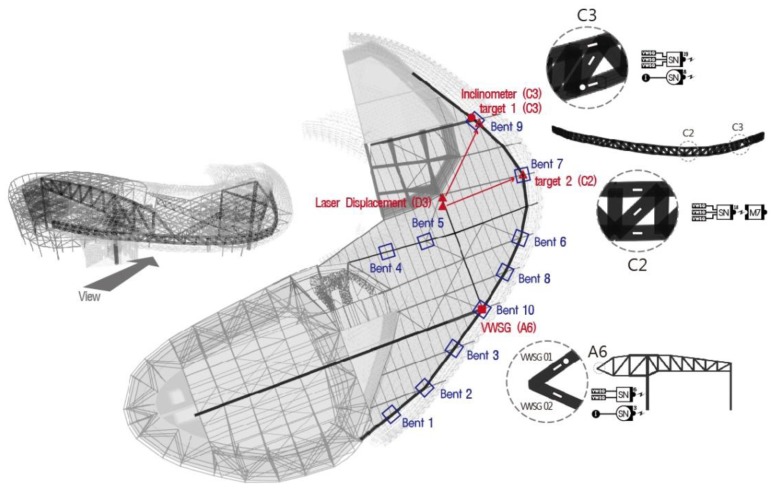
Measuring points and bent locations (Bent No. indicates the order of bent removal.).

**Figure 12.**
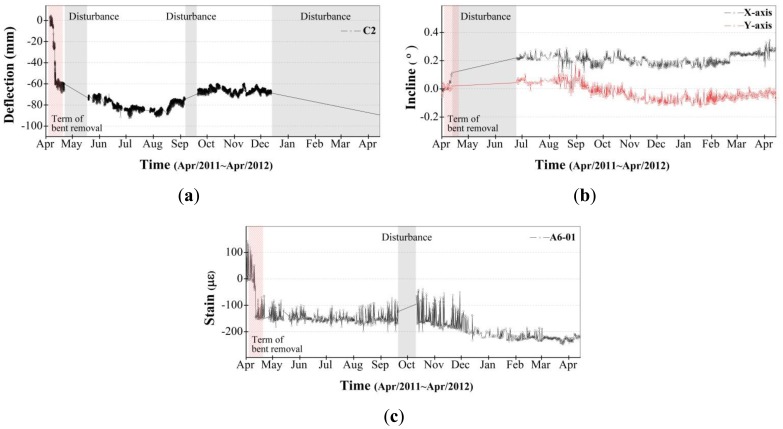
13-month monitoring. (**a**) Deflection in zone C2 (**b**) Inclination in zone C3; (**c**) Strain data in zone A6-01.

The locations for all measured data are provided in Figure 11. Figure 12 illustrates the change over time in the data measured during the 13 months from April 2011 through April 2012. Figure 12a is the vertical deflection measured by the laser displacement sensor installed in the zone C2 edge truss, Figure 12b is the inclination measured from inclinometer installed in zone C3, and Figure 12c is the strain measured by a VWSG in the zone A6 mega truss. As shown in the disturbance period in Figure 12, not all of the data were acquired during the 13-month monitoring period. This fact may be due to measurements being disturbed by communication obstacles, indicating the difficulty in monitoring a structure under construction, where many obstacles and unexpected events can arise.

The edge truss in zone C that is connected to the mega truss is a cantilevered structure; before completing the process of welding the separately manufactured mega truss, it was supported by 10 temporary bents arranged in equal intervals along the length of zone C (Figure 11).

After the welding connections of the fabricated mega truss and edge truss were completed, the temporary bents were removed, as shown in Figure 11. As these bents were removed (20–25 April 2011, indicated as the “term of bent removal” in Figure 12), a vertical deflection occurred at the edge truss and mega trusses due to the weight of the structure that had been supported by the bents. This construction event (*i.e.*, bent removal) exhibited the largest influence on the responses of the structural members during the monitoring period.

**Figure 13.**
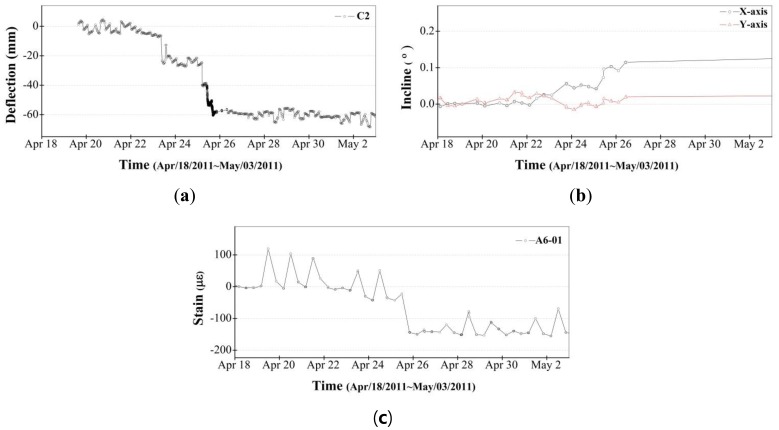
Terms of bent removal. (**a**) Deflection in zone C2; (**b**) Inclination in zone C3; (**c**) Strain data in zone A6-01.

Figure 13 presents the measured data for 17–26 April 2011, which focuses on the behavior of the structural members during the bent removal event. The largest change occurred on the 25 April 2011, when the final bent was removed. The location of the bent removed on that day was the point (bent 9 in Figure 11) connecting the mega truss in zone B to the edge truss in zone C, and the point (bent 10 in Figure 11) connecting the mega truss in zone A to the edge truss in zone C. Therefore, because the cantilever behavior (*i.e.*, vertical deflection at the free end) began in the mega truss and edge truss, it had the largest effect on the measured values. Because A6 and C3 are the connecting areas of the mega truss and edge truss, they were areas that required great caution in terms of structural safety when planning the SHM system for DPB. Therefore, the measuring interval of the laser displacement sensors during the bent removal process was changed from 30 min to 1 min to monitor the amount of deflection in real time.

Figure 13a illustrates the vertical deflection measured at point C2, where the largest vertical deflection was measured among the three target points (C2, C3 and A6 in Figure 11). Point C2 is located between points C1 and C3, which are supported by the mega trusses. This largest deflection was attributed to not only the deflections induced from cantilever behaviors of mega trusses but also the deflection at the center of the edge truss after bent removal. In a quantitative evaluation, the maximum deflection of 67 mm at point C2 after bent removal was smaller than the initial camber values determined from the predicted vertical deflection of 164.4 mm, from which the safety of the edge truss during construction can be assured.

Figure 13b illustrates the variation of inclination along the X and Y axes measured at point C3 of the edge truss (Figure 11). The X and Y axes indicate the longitudinal and transverse directions of the edge truss, respectively. The inclination along the X axis represents the ratio of the difference in the vertical deflections (54 mm) that were measured at points C2 (67 mm) and C3 (13 mm) to the distance (approximately 30 m) between points C2 and C3, and the inclination along the Y axis indicates the ratio of the vertical deflection at point C3 to the cantilever length of mega truss B (14.1 m). A comparison between the inclinations of the X and Y axes demonstrates that the variation of inclination along the X axis during bent removal is larger than that along the Y axis. This observation can be attributed to the fact that the largest deflection occurred at point C2 and the smallest deflection occurred at point C3 of mega truss B.

Figure 13c illustrates the variations in strain rates resulting from the data collected three times a day (once every 8 h). Although the three measurements were collected on a single day, there is a substantial disparity in their values that may be attributable to the influence of temperature stress caused by the wide variation in daily temperatures between the morning and afternoon periods. A substantial change occurred on 25 April 2011, when all bents were removed. The negative value in strain shown in Figure 13c indicates a tensile state that resulted from the vertical deflection at the free end of the mega truss after bent removal. The ratio of maximum strain value (measured after all bents were removed) to the yield strain is approximately 15%. Thus, the safety of the edge truss and mega truss could be confirmed from the recorded response data.

Based on the bent removal monitoring results, we can obtain not only the local response of the members (strain) but also the global responses (deflection and inclination) of the structure, thereby evaluating the overall safety of the structure. Furthermore, it is also confirmed that the relationships between these responses can provide information on the global behavior of the structure induced from a specific event (e.g., the vertical deflection in this study).

## 5. Conclusions

This research developed a practical and systematic SHM system that can be applied to a structure under construction, where many challenging issues exist. In this system, customized energy-efficient wireless sensing units (sensor nodes, repeater nodes, and master nodes) were employed and comprehensive communications from the sensor node to the monitoring server (*i.e.*, sensor node–repeater node-master node-monitoring server) were conducted through wireless communications by using the ISM band and CDMA method, which were responsible for short- and long-distance communications, respectively. Through these decentralized communications, energy-efficient communication devices operated by battery power were enabled. As a result, wireless communications employed at a construction site drastically reduced cable lengths—a major issue related to in-construction monitoring.

The monitoring system proposed here was applied to a large-scale irregular building under construction that exhibited a very complicated configuration and long span (approximately 118 m) supported by cantilevered edge truss and mega trusses. The long-term monitoring results recorded from a large number of sensors (such as VWSGs, inclinometers, and laser displacement sensors) indicated that the construction event exhibiting the largest influence on structural behavior was the removal of bents that were temporarily installed to support the free end of the cantilevered members (such as the edge truss and mega trusses) during their construction. Bent removal caused a significant vertical deflection of the edge truss and the mega trusses induced from the cantilever behavior. The safety of each member could be confirmed based on the quantitative evaluation of each response. Furthermore, it was also confirmed that the relation between these responses (*i.e.*, deflection, strain, and inclination) can provide information about the global behavior of structures induced from specific events (e.g., the vertical deflection in this study).

Analysis of the long-term (13-month) measurement results demonstrates the proposed sensor network system is capable of automatic and real-time monitoring and can be applied and utilized for both the safety evaluation and precise implementation of buildings under construction. Furthermore, the measured values obtained through this construction monitoring technique can be utilized as fundamental information when analyzing and evaluating the response of a structure subjected to the various loads that occur during the building's service period. This ability is very important for providing continuous information on the construction stage in terms of understanding the behavior of a structure in-service.

Finally, we observed the limitations in the proposed WSN throughout the long-term monitoring, including the data loss caused by communication obstacles at the construction site. Therefore, the development of data recovery tools in terms of both hardware and software (e.g., such as signal processing methodology) is needed to enhance the reliability of monitoring results with data loss. In addition, since the proposed system is applied to measure the static responses in this study, the capability of measuring dynamic response such as acceleration is recognized as an important issue involving stable long-term power supply, which should be resolved to enhance its applicability.

## Figures and Tables

**Table 1.** Device specifications.

**Parameter**	**Sensor**			**Sensor Node**	**Master Node**

	**VWSG**	**Inclinometer**	**LDS**		
Measurement Range	300 με	−15°∼15°	0.2∼35 m	550∼6,000 Hz (10,000 με or more)	
Degree of Precision	1 με	0.0025°	±1 mm	1 με	
Operating Temperature Range	−20∼80 °C	−30∼60 °C	−10∼60 °C	−30∼85 °C	
Communication Range	wired connection to sensor node			400 m (LOS *), 100 m (NLOS **)	-

* LOS: Line of Sight;

** NLOS: Non-Line of Sight.
